# Persistence of IgM Antibodies after Vaccination with Live Attenuated Japanese Encephalitis Vaccine

**DOI:** 10.4269/ajtmh.20-1132

**Published:** 2020-11-23

**Authors:** Susan L. Hills, Alex Van Keulen, Jodi Feser, Amanda Panella, G. William Letson, J. Erin Staples, Anthony A. Marfin, Aaron C. Brault

**Affiliations:** 1 Arboviral Diseases Branch, Centers for Disease Control and Prevention, Fort Collins, Colorado;; 2 PATH, Seattle, Washington

## Abstract

Japanese encephalitis (JE) is a vaccine-preventable, mosquito-borne disease. Substantial progress with JE control in Asia has been made during the past decade, with most endemic countries now having JE vaccination programs, commonly using live attenuated SA14-14-2 JE vaccine (trade name CD-JEV). If a child develops encephalitis during the weeks to months following CD-JEV vaccination and anti-JE virus IgM (JE IgM) antibody is detected in serum, the question arises if this is JE virus infection indicating vaccine failure, or persistent JE IgM antibody postvaccination. To better understand JE IgM seropositivity following vaccination, sera from 268 children from a previous CD-JEV study were tested by two different JE IgM assays to determine JE IgM antibody frequency on days 28, 180, and 365 postvaccination. With the CDC JE IgM antibody capture ELISA (MAC-ELISA), 110 children (41%) had JE IgM positive or equivocal results on their day 28 sample, and eight (3%) and two (1%) had positive or equivocal results on day 180 and day 365 samples, respectively. With the InBios JE *Detect™* MAC-ELISA (Seattle, WA), 118 (44%) children had positive or equivocal results on day 28 sample, and three (1%) and one (0.4%) had positive or equivocal results on day 180 and day 365 samples, respectively. Our results indicate that more than 40% children vaccinated with CD-JEV can have JE IgM antibodies in their serum at 1 month postvaccination but JE IgM antibody is rare by 6 months. These data will help healthcare workers assess the likelihood that JE IgM antibodies in the serum of a child with encephalitis after vaccination are vaccine related.

## INTRODUCTION

Japanese encephalitis (JE) is a vaccine-preventable, mosquito-borne disease found in Asia and parts of the western Pacific. Fewer than 1% of persons infected with JE virus develop neurological illness, but when disease occurs, it can be severe with a 20–30% mortality rate and 30–50% of survivors left with long-term sequelae. Substantial progress with JE control has been made during the past decade, and most JE-endemic countries now have JE vaccination programs. The live attenuated SA14-14-2 JE vaccine (trade name CD-JEV) produced by Chengdu Institute of Biological Products in China is the vaccine used most frequently in these programs.^1^

The typical IgM antibody pattern observed in viral infections is that it appears in serum during the acute phase of infection and falls to non-detectable levels within 60–90 days.^2^ However, it is well recognized that long-term persistence of IgM antibody can occur following some viral infections. IgM persistence in serum has been documented following many flaviviral infections, including with West Nile virus for > 7 years, Zika virus for > 2 years, JE virus for up to 1 year, and dengue virus for > 1 year in some subjects in cohort studies.^3–9^ Similarly, following administration of yellow fever vaccine, a live attenuated flaviviral vaccine, to U.S. residents who had no evidence of infection with yellow fever virus or related flaviviruses before vaccination, almost three-quarters (73%; 29 of 40) of individuals had yellow fever IgM antibodies 3–4 years later.^10^

There are limited data on the detection of anti-JE virus IgM (JE IgM) antibody in serum following vaccination with JE vaccines. In a study among Korean children aged 1–3 years vaccinated with CD-JEV, nine (13%) of 68 children had JE IgM antibody detected 4 weeks after vaccine administration.^11^ In another study in Korea with 14 children vaccinated with two doses of CD-JEV at a 12-month interval, none had IgM antibody detected at a mean of 21 months (range: 3–47 months) after the second dose.^12^ Pre-vaccination serum was not collected in either study to determine if the children were immunologically naive to JE virus or another flavivirus infection at the time of vaccination, which could have affected the immune response and study results. In a study using an experimental live recombinant JE vaccine, constructed by replacing genes encoding the pre-membrane and envelope proteins of yellow fever 17D vaccine virus with the corresponding JE virus genes, nine (75%) of 12 yellow fever immune adults and all 12 nonimmune adults had JE IgM antibodies at approximately 1 month postvaccination.^13^ Among adults vaccinated with a two-dose schedule of an inactivated, Vero cell–derived JE vaccine, 33 (33%) of 100 had detectable IgM at some point during 28–56 days following the second dose, including 15 (15%) of 97 with IgM detectable on day 56.^14^ Finally, IgM production following vaccination with mouse brain–derived JE vaccine also has been reported.^15,16^

CD-JEV has been used extensively in mass vaccination campaigns and routine infant vaccination programs in Asia. If a child develops encephalitis during the weeks to months following CD-JEV vaccination, and JE IgM antibodies are detected in serum in the absence of a diagnostic cerebrospinal fluid (CSF) sample, the question arises whether the serum IgM suggests a wild-type JE virus infection indicating vaccine failure or reflects postvaccination IgM persistence. Understanding the frequency and duration of IgM antibodies in serum following vaccination would be useful in these circumstances. We conducted testing with two JE IgM assays to determine the frequency of JE IgM antibody seropositivity at several time points following vaccination with CD-JEV.

## MATERIALS AND METHODS

### Samples.

Archived serum samples from a 2007–2008 clinical trial to assess CD-JEV safety and immunogenicity were used.^17^ The previous study was conducted in the district of Colombo, Sri Lanka. Healthy children were coadministered CD-JEV and measles vaccines at the age of 9 months (±2 weeks). Sera were collected on day 0 before vaccination and on days 28, 180, and 365 postvaccination. Reported seroprotection rates, based on a JE virus neutralizing antibody titer of ≥ 1:10, were 91% (233 of 257) on day 28 postvaccination and 87% (221 of 253) on day 365, confirming adequate vaccine immunogenicity.^17,18^ After completion of neutralization testing, serum samples were stored for approximately 10 years at −20°C at a research facility in Thailand. These samples were shipped in 2019 to the Arboviral Diseases Diagnostic and Reference Laboratory at the U.S. CDC (Fort Collins, CO) where they were stored at −20°C until testing.

### Laboratory testing.

Sera were tested at the CDC with two JE IgM antibody capture ELISAs (MAC-ELISAs). One was the in-house CDC JE MAC-ELISA previously developed at the CDC’s Arboviral Diseases Diagnostic and Reference Laboratory.^19^ The other was the commercial InBios JE *Detect* MAC-ELISA (Seattle, WA), which is commonly used by public health laboratories in JE-endemic countries in Asia; in different evaluations, the assay’s sensitivity for use with sera ranged from 57% to 100%.^20–22^ For the CDC MAC-ELISA, a positive-to-negative (P/N) value < 2.0 was interpreted as negative, a P/N from 2.0 to < 3.0 was equivocal, and a P/N ≥ 3.0 was positive; a nonspecific reaction could also be reported. For the InBios assay, results were interpreted as positive, negative, or equivocal according to directions in the product insert.^23^ Initially, all day 28 samples were tested. If a subject’s day 28 sample had an IgM positive or equivocal result with the assay in use, the day 180 sample was tested, and if the day 180 sample had a positive or equivocal result, the day 365 sample was tested. Testing was conducted sequentially. If a sample tested negative at one time point with a particular assay, it was assumed that all subsequent samples from that participant also would be negative with that assay. If a result from a specific time point was unknown because the sample was unavailable, sample volume was insufficient for testing, or the CDC MAC-ELISA produced a nonspecific result, the sample from the subsequent time point was tested.

### Data management and analysis.

Each subject’s baseline, pre-vaccination level of JE virus neutralizing antibody was gathered from data files from the previous study, in which a 50% plaque reduction neutralization test was used and subjects were considered seroprotected if their neutralizing antibody titer was ≥ 1:10.^17,18^ Data were analyzed using Excel version 2016 (Microsoft, Redmond, WA). CIs were calculated using the exact method. The denominator for proportion positive at each time point was kept constant at 268 children who had at least one postvaccination sample tested. Unknown results (i.e., the child did not have a sample available or sufficient for testing or the sample had a nonspecific result) were included with negative results when calculating the percent of samples positive. The larger clinical trial protocol, including this component, was approved by PATH’s Research Ethics Committee, USA, and the University of Colombo Faculty of Medicine Ethical Review Committee.^17^ The secondary laboratory analysis described here was also reviewed at the CDC in accordance with standard CDC procedures, which determined the work was outside the scope of Institutional Review Board review requirements as it was not considered to involve human subjects.

## RESULTS

A total of 268 subjects had samples available. On testing with the CDC MAC-ELISA, at day 28, 46 (17%; 95% CI: 13–22) of 268 children had positive IgM test results and 64 (24%; 95% CI: 19–29) had IgM equivocal test results for their sample (Table 1). At day 180, eight (3%; 95% CI: 1–6) children had positive or equivocal test results, and at day 365, two (1%; 95% CI: 0.1–3) had positive (*n* = 1) or equivocal (*n* = 1) results. With the InBios JE *Detect* MAC-ELISA, at day 28, 75 (28%; 95% CI: 23–34) of 268 children had IgM positive test results and 43 (16%; 95% CI: 12–21) had IgM equivocal results; at day 180, three (1%; 95% CI: 0.2–3) had positive or equivocal results; and at day 365, one (0.4%; 95% CI: 0.01–2) had a positive result. Overall, when combining positive and equivocal results at day 28, 110 (41%; 95% CI: 35–47) and 118 (44%; 38–50) children had positive or equivocal results when their samples were tested with the CDC MAC-ELISA or JE *Detect* MAC-ELISA, respectively.

**Table 1:** Japanese encephalitis (JE) IgM antibody test results with the CDC JE IgM antibody capture ELISA (MAC-ELISA) and InBios JE *Detect* MAC-ELISA for serum samples collected on days 28, 180, and 365 following vaccination of 268 children with live attenuated SA14-14-2 JE vaccine

Time postvaccination	JE IgM result	CDC assay	InBios assay
		No. (%)	No. (%)
Day 28			
	Positive	46 (17)	75 (28)
	Equivocal	64 (24)	43 (16)
	Unknown	15* (6)	10** (4)
	Negative	143 (53)	140 (52)
Day 180†			
	Positive	2 (1)	2 (1)
	Equivocal	6 (2)	1 (0.4)
	Unknown	9‡ (3)	11¶ (4)
	Negative	108 (40)	114 (43)
	Presumed negative#	143 (53)	140 (52)
Day 365†			
	Positive	1 (0.4)	1 (0.4)
	Equivocal	1 (0.4)	0 (0)
	Unknown	1§ (0.4)	0 (0)
	Negative	14 (5)	13 (5)
	Presumed negative#	251 (94)	254 (95)

* Ten subjects had no sample, and five samples had a nonspecific reaction.

† Samples were tested if results were positive, equivocal, or unknown with the relevant assay at previous time point.

‡ Nine subjects had no sample.

§ One subject had no sample.

** Ten subjects had no sample.

¶ Eleven subjects had no sample.

# If a sample tested negative at a previous time point, it was presumed all subsequent samples from that participant also would be negative with that assay.

Data from the previous study indicated 15 (6%) children had a seroprotective neutralizing antibody titer on day 0. On testing their day 28 samples, seven (47%) of 15 had no detectable IgM antibody with either assay; one (7%) had equivocal IgM results with both assays; one (7%) had positive IgM results with both assays; five (33%) had a positive result with the InBios assay and an equivocal (*n* = 3), nonspecific (*n* = 1), or negative (*n* = 1) result with the CDC assay; and one (7%) had no day 28 sample for testing.

## DISCUSSION

At 1 month after vaccination with CD-JEV, > 40% of children had a JE IgM positive or equivocal result on testing their serum, suggesting that if a child in a JE-endemic area presents with encephalitis, has JE IgM antibody in serum, and recently has been vaccinated with CD-JEV, consideration should be given to the antibody being vaccine related, and alternate etiologies of encephalitis should be sought. However, by 6 months postvaccination, < 5% of children had detectable IgM in serum. Therefore, at ≥ 6 months postvaccination, JE IgM antibodies detected in serum have a higher likelihood of being disease related.

Testing conducted using the two MAC-ELISAs produced a relatively similar percentage of samples with positive and equivocal results at each time point. At day 28, the JE *Detect* MAC-ELISA had a somewhat higher percentage of positive results (28%) and lower percentage of equivocal results (16%) than the CDC MAC-ELISA (17% positive and 24% equivocal); however, when positive and equivocal results were combined, 41% and 44% of samples had positive or equivocal results with the two assays, respectively.

Among the 15 children with a seroprotective JE neutralizing antibody titer on day 0 before vaccination, almost 50% had positive or equivocal JE IgM antibody results with at least one of the assays at day 28 postvaccination, similar to results among the children with no detectable neutralizing antibodies before vaccination. Whether the detected neutralizing antibodies were of maternal origin or because of previous JE virus exposure in the infant is unknown. However, these results suggest the presence of neutralizing antibodies at the time of vaccination did not prevent some children forming IgM antibodies and mounting an immune response to vaccination.

The JE IgM antibody detected could possibly represent cross-reactive antibody from recent dengue or other flavivirus infections. However, we did not conduct additional testing for other infections as our study objective was to assess the frequency of detectable JE IgM antibody postvaccination in a true field setting where other flaviviruses might be circulating. It is unlikely a substantial proportion of the JE IgM antibody detected represented cross-reactive antibody. Unless a flavivirus outbreak occurred specifically around the time when the day 28 samples were being collected, a higher rate of JE IgM positivity than was observed would have been expected in samples from the day 180 and day 365 time points, resulting from cross-reactive antibody to the other flavivirus. Nonetheless, if a considerable proportion of the JE IgM antibody truly represented cross-reactive antibody, study findings would be different in a setting with different levels of transmission of other flaviviruses. For diagnostic purposes, in areas with transmission of JE and dengue viruses, anti-dengue virus IgM testing should routinely be conducted on serum samples with JE IgM antibody.^22^ In addition, WHO laboratory guidance recommends confirmatory testing on receipt of a positive JE IgM antibody result in various situations, and the WHO has designated several laboratories within its JE laboratory network as reference laboratories to provide such testing.^24,25^

Our study has several limitations. The proportion positive or equivocal at each time point might be slightly underestimated because a number of samples were not available or had nonspecific results, and these results were included with negative results for the purpose of determining percent sample positivity. Samples had been stored for 10 years before IgM testing was conducted, and any antibody degradation and resultant impact on detection of IgM antibody is unknown. In the original study, CD-JEV was coadministered with measles vaccine, and it is unknown if this might have had any impact on the development of JE IgM antibody. Natural JE virus infection as the cause of the IgM cannot be excluded, although the study was conducted in children from low JE endemicity peri-urban areas in the district of Colombo. All subjects were aged 9 months at the time of vaccination, so we cannot be certain that similar findings pertain to children vaccinated at different ages (e.g., aged 12 months or older if JE vaccine is administered in mass vaccination campaigns or routine programs using a target age-group older than 9 months). Finally, no samples were collected between day 28 and day 180, so no information is available on the rate of JE IgM antibody detection between these two time points.

Detection of IgM antibody in the CSF is considered the most reliable method for JE diagnosis.^26^ If JE IgM antibody is detected in the CSF without peripheral blood contamination, it denotes infection of the central nervous system because IgM is unable to cross the blood–brain barrier, and indicates JE virus as the cause of the patient’s encephalitic illness.^27^ Although optimal clinical management of a patient with suspected encephalitis includes a lumbar puncture (LP) unless there are contraindications, LPs are not routinely conducted in some JE-endemic areas, and serum alone is often used as the basis for JE diagnosis.^27^ Our results will help healthcare workers determine whether the presence of JE IgM antibodies in serum of a child with encephalitis in the weeks to months after CD-JEV vaccination are because of persistent IgM antibody following vaccination or possibly from a JE virus infection that occurred because of JE vaccine failure.
